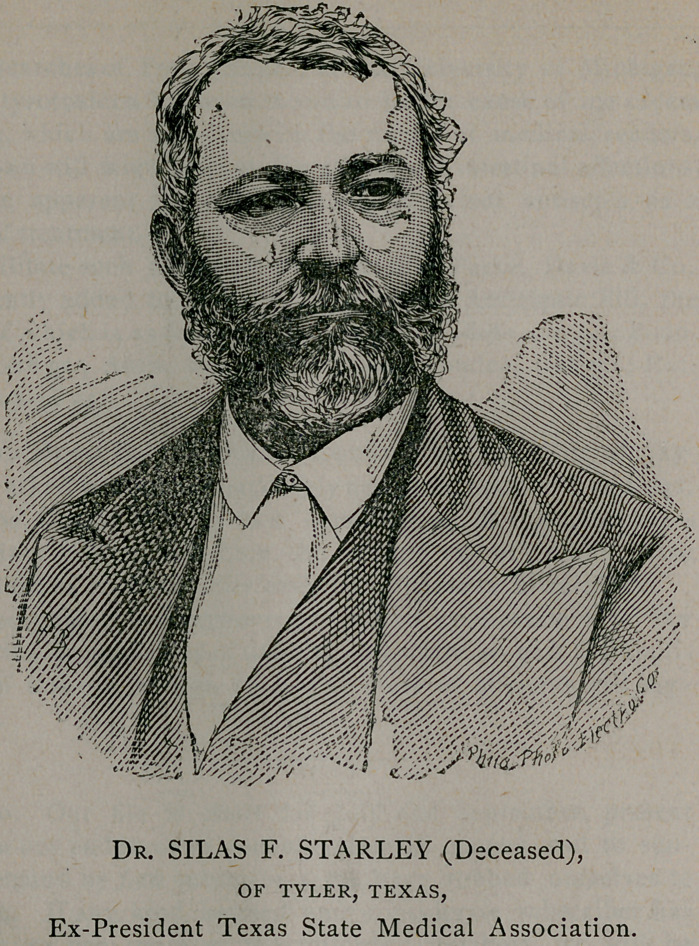# Dr. Silas F. Starley

**Published:** 1888-02

**Authors:** 


DR. STARLEY was born in Autauga county, Alabama, September 5, 1824, and was 63 years of age at the time of his death, December 19, 1887. He was a graduate of the Medical Department University of Louisville, Ky. His entire professional life was spent in Texas. In 1882 Dr. Starley was elected President of the Texas State Medical Association, at the Fort Worth convention, and presided as such at Belton, the following year. His biography was published in the JOURNAL last issue.

## Figures and Tables

**Figure f1:**